# A Review of “Access to Care” Issues in Aortic Stenosis Patients: A Negative Report Card

**DOI:** 10.1016/j.shj.2024.100351

**Published:** 2024-08-17

**Authors:** Adam M. Reisman, Sammy Elmariah

**Affiliations:** a Mount Sinai Fuster Heart Hospital, Icahn School of Medicine at Mount Sinai, New York, New York; b Division of Cardiology, Department of Medicine, University of California San Francisco, San Francisco, California

**Keywords:** Access to care, Aortic stenosis, Aortic valve disease, Aortic valve replacement, Health disparities, TAVR

## Abstract

The identification and management of patients with aortic stenosis exist along a continuum that includes healthy living, latent progression, diagnosis, treatment, and posttreatment recovery. Barriers to the provision of appropriate care for these patients can occur at any stage along this continuum. Despite the presence of diagnostic echocardiograms, many patients with aortic stenosis are never clinically recognized, and the rate of mismanagement worsens among underrepresented minority groups and women. Regarding the treatment of clinically recognized aortic stenosis, only about half of patients with symptomatic severe aortic stenosis actually undergo aortic valve replacement within 2 ​years of diagnosis. Treatment rates are even lower among patients with symptomatic low-gradient severe aortic stenosis. Although several strategies have been raised by experts within the field to help and improve the diagnosis and treatment of patients with aortic valve disease, timely referral to a heart valve team specialist whenever aortic valve replacement is being considered likely remains the most pertinent intervention. Connecting these patients with fully informed aortic valve disease experts helps facilitate shared decision-making discussions, thus ensuring that patients have the opportunity to learn about and potentially receive the lifesaving interventions available to them.

## Highlights

• One in four patients with moderate or severe aortic stenosis (AS) is not clinically recognized.
• Rates of missed diagnoses worsen among underrepresented minority groups and women.
• Only 50% of patients with symptomatic severe AS undergo aortic valve replacement (AVR) within 2 years.
• One in three patients with symptomatic high-gradient severe AS does not receive AVR.
• Two in three patients with symptomatic low-gradient severe AS do not receive AVR.

## Introduction

Aortic stenosis (AS) should be thought of as a continuum that includes healthy living, latent progression, diagnosis, treatment, and posttreatment recovery. Barriers to care can occur at any stage along this continuum. The decision to pursue treatment such as aortic valve replacement (AVR) is typically triggered by several clinical factors, including the patient’s symptom status, severity of AS, left ventricular function, or whether other invasive cardiac interventions are already indicated.^1^ In cases where AVR is clinically indicated but not pursued, the literature has shown that these patients are at increased risk of aortic valve disease progression, hospitalization, and death. These trends are consistent among both symptomatic and asymptomatic severe AS patients, as well as those with reduced left ventricular function.^2^, ^3^, ^4^

## Diagnostic Disparities

To assess the rate at which clinicians recognize and formally diagnose new AS, Crousillat et al. analyzed patient records within the Mass General Brigham health care system from 1990 to 2020. Patients with any degree of AS on transthoracic echocardiogram (TTE) were identified, excluding those with a pre-existing diagnosis code for AS (International Classification of Diseases [ICD]-9 or ICD-10), and the earliest date of diagnostic billing code assignment for AS was determined. Self-reported racial and ethnic data were used to assess whether disparities existed within the diagnostic process.^5^

The study population included 14,800 patients, and the AS severity across the population was 81.8% mild, 13.6% moderate, and 4.7% severe. Within 1 year of echocardiographic evidence of moderate AS, only 71.0% of physicians assigned an ICD code of AS. For those found to have severe AS, the rate of ICD code assignment was 84.7% within 1 year. Given the increased mortality risk associated with severe AS, this rate of diagnosis is suboptimal. Additionally, despite similar proportions of patients within each severity class across racial and ethnic groups, the rates of diagnosis among Black and Hispanic patient populations were lower than those of White patients. White patients with moderate AS had a diagnosis rate of 71.5%, while the diagnosis rate of Black and Hispanic patients was only 60.8 and 62.5%, respectively. More concerning, among patients with severe AS, White patients had a diagnosis rate of 85.8%, while that of Black and Hispanic patients was only 61.1 and 66.7%, respectively. This gap in care between racial and ethnic groups may be even greater when accounting for those who never underwent an echocardiogram in the first place.^5^

Several independent predictors of whether patients with AS receive an appropriate ICD diagnosis were identified. Increased AS severity was associated with increased recognition and diagnosis. Women were 20% less likely to receive a diagnosis. Non-Hispanic Black and Asian patients were 35 and 28%, respectively, less likely than non-Hispanic White patients to receive a diagnosis. Importantly, TTEs ordered by noncardiology providers or obtained within the inpatient setting led to reduced AS recognition. Given the higher proportion of minority patients within the inpatient setting, this last point is of particular concern with respect to racial and ethnic disparities. Ultimately, poor transitions from inpatient to outpatient care, barriers to cardiology-specific care, and barriers to higher levels of care all significantly impact the management of aortic valve disease.^5^

## Undertreatment

The use of AVR for patients with severe AS was analyzed by Li et al. via patient data from Mass General Hospital and Brigham and Women’s Hospital from 2000-2017. This study identified 10,795 patients with severe AS, defined as aortic valve area less than 1 cm^2^, who were further broken down by high-gradient vs. low-gradient AS, low ejection fraction (EF) vs. normal EF, and symptomatic vs. asymptomatic. High vs. low gradient AS was defined as a mean aortic valve gradient ≥40mmHg vs. <40mmHg, respectively. Low vs. normal EF was defined as <50% vs. ≥50%, respectively. Natural language processing models were developed and validated to identify symptoms consistent with severe AS, AS-related referrals, and AVR refusals.^6^

Among patients with symptomatic high-gradient AS with a normal EF, a class I indication for AVR, only 70% received AVR. Taking symptoms out of the equation (i.e., removing the natural language processing model), among patients with high-gradient AS and a low EF, also a class I indication for AVR, only 53% underwent AVR within a 2-year period. Within the symptomatic low gradient cohort, a potential class IIa indication for AVR, treatment rates were even lower. Only 32% of the low gradient-normal EF group and 38% of the low gradient-low EF group underwent AVR, likely reflecting an incomplete understanding of the low-gradient phenotypes of severe AS. Women with low-gradient AS had 40% lower odds of receiving AVR, which is especially troubling given that women are more likely to have lower gradients. Increased patient age was also associated with significant underutilization of AVR, as only one in four patients aged greater than 80 years with severe symptomatic AS received AVR. In summary, contributors to the underutilization of AVR included low mean gradient, severe AS, older age, female sex, inpatient TTE, low left ventricular EF, and low hematocrit. Patients who were more likely to undergo AVR included smokers and those with coronary artery disease. Overall, less than 50% of patients with symptomatic severe AS that had a guideline-driven indication for AVR were appropriately treated. This outcome is of significant importance given that AVR was associated with a decreased adjusted hazard of mortality of 27% among low gradient-normal EF patients, 52% among low gradient-low EF patients, 58% among high gradient-normal EF patients, and 72% among high gradient-low EF patients.^6^

As to why AVR is deferred for certain patients, Flannery et al. analyzed ambulatory patients within the Massachusetts General Hospital echocardiographic database demonstrating severe AS. This patient group was a general cohort including any patient who underwent TTE between April 2016 and April 2018 with an aortic valve area ≤1.0 cm^2^. Of 324 patients with an indication for AVR, 140 patients (43.2%) did not undergo AVR. Reasons for lack of treatment included lost to follow-up, watchful waiting for symptoms to worsen, AS not felt to be severe, symptoms not attributed to AS, patient decline, medical futility, AS evaluation underway, and no evidence of AS evaluation. Nowhere in the current clinical guidelines does it state that symptoms need to be severe before performing AVR. Regarding patients in whom their AS was not felt to be severe, this likely represents an underappreciation of the low-gradient phenotypes, as previously mentioned. “Patient declined” and “medical futility” made up 11% and 7%, respectively, of patients not getting AVR. Twenty percent of patients had an AS evaluation started but not completed. Ten percent of patients did not have any evidence of their AS being acted upon whatsoever.^7^

Importantly, no cases of medical futility or patient declining intervention occurred in conjunction with a heart valve team specialist evaluation. Decisions not to pursue treatment were largely made via discussions with nonvalve team members, some of whom may have been cardiologists but often were primary care physicians. As reported by the American College of Cardiology/American Heart Association in 2020, the “choice of TAVI (transcatheter aortic valve implantation) versus palliative care is based on a decision-making process that accounts for the patient’s values and preferences and includes discussion of the indication, risks, and benefits for and against each approach.”^8^ Thus, referral to a heart valve team specialist is critical in order to allow for a fully informed discussion regarding the natural history of aortic valve disease and the specifics of interventions under consideration. If stakeholders leading that discussion lack the necessary level of expertise, the result, as has been demonstrated by the data, is suboptimal management of valvular heart disease.

## Opportunities for Improvement

In addition to identifying the problems of underdiagnosis and undertreatment among aortic valve disease patients, we must also discuss potential solutions to these problems. One possibility for improvement takes advantage of the electronic medical record (EMR). We have seen the benefit of the EMR with respect to facilitating patient recruitment for enrollment in clinical trials.^9^ It is therefore reasonable to suggest that tools within the EMR could be leveraged to enable the recognition of patients with AS and subsequently notify the appropriate providers, thus expediting timely and appropriate disease management.

The DETECT AS trial (ClinicalTrials.gov ID: NCT05230225) is a randomized clinical trial that identifies patients in whom echocardiography shows severe AS and then randomly assigns the ordering providers to the EMR intervention vs. no intervention. Providers assigned to the EMR intervention group receive an electronic notification within the EMR for each of their patients with severe AS on TTE, and are also provided with the relevant American College of Cardiology/American Heart Association Clinical Practice Guidelines recommendations regarding the management of severe AS. The purpose of this study is to investigate the impact of electronic provider notification on the management of severe AS, the utilization of AVR, and the ethnic and racial disparities in AVR utilization.^10^

Another opportunity for improvement may lie within the echocardiogram reports themselves. These reports can at times be challenging for referring physicians to understand or apply clinically, and therefore may lead to an incomplete follow-up of relevant findings.^11^ Perhaps it is insufficient to simply report echocardiographic results without also detailing a clear clinical assessment and plan. Explicit clarification of potential critical findings becomes especially important when the data presented are discordant. For example, in patients with low-gradient severe AS (as opposed to those with high gradients), providers (whether they are general practitioners, cardiologists, or other specialists) may be less familiar with the nuances of AS diagnosis and management. One could argue the need for increased educational initiatives among physicians regarding the urgency of aortic valve disease, appropriateness of referrals, and the importance of initiating the treatment pathway, but the success of educational initiatives may be unrealistic for a profession that is already barraged with information and has increasingly limited available time per patient. Instead, we may need to address echocardiographer reporting standards and encourage the inclusion of clearly delineated next steps/follow-up. National standards are commonly seen across other cardiac disease processes such as ST elevation myocardial infarction (i.e., door-to-balloon time) and cholesterol levels. Aortic valve disease should be no different.

Lindman et al. have laid some of the groundwork to help improve outcomes among AS patients through the “Target Aortic Stenosis” pilot initiative. This initiative assessed the clinical processes that occur following initial echocardiographic evidence of AS in order to develop a means of systematically evaluating quality of care prior to receiving definitive AS treatment. Recommended quality metrics included measurement of the percentage of patients with a class I indication for AVR who have been treated within 90 days of their diagnostic echocardiogram, the percentage of echocardiograms with AS that consistently reported pertinent physiologic metrics (e.g., aortic valve area, transvalvular mean gradient, transvalvular peak gradient, stroke volume index, and left ventricular ejection fraction) as well as a qualitative description of AS severity, the percentage of echocardiograms showing moderate or severe AS that have a follow-up echocardiogram within 24 or 12 months (respectively), the percentage of patients treated with AVR who were seen by a multidisciplinary heart valve team prior to their procedure, and the percentage of patients with a class I indication for AVR who were treated within 30 days of the multidisciplinary heart valve team evaluation.^12^ Formalizing the way in which we measure quality of care after the diagnosis of AS may help address some of the treatment disparities that currently exist based on sex, race, and ethnicity, as described above.

Lastly, the reimbursement for AVR often varies across different patient groups as well as across different geographies. Studies have shown that Medicare reimbursement rates based on geography alone can range from $32,000 to over $60,000 for a transcatheter aortic valve replacement (TAVR) procedure for clinically equivalent patients.^13^ There are health care facilities throughout the country that lose money for each AVR that is performed, despite clinical appropriateness of intervention. A 2017 study assessing hospital revenues following AVR found that the median TAVR contribution margin, defined as a hospital’s revenue minus variable cost per specific procedure performed, was net negative $3380.^14^ Hospitals in which these procedures were performed at a net loss tended to serve more minority patients or patients of lower socioeconomic status.^15^ Furthermore, it has been shown that among hospitals with existing cardiac surgery capabilities, those that never established TAVR programs were more likely to be located in the South, the region with the highest proportion of minority patients.^16^ This clearly limits the provision of care to patients in need and happens to disproportionately impact minority groups, thus worsening racial and ethnic disparities in the provision of care. Addressing issues of reimbursement with both federal and private payors on a national level may help to alleviate this issue.

## Limitations

It is important to note that while the abovementioned studies offer a high degree of evidence regarding the barriers to care that exist in the diagnosis and management of AS, there are limitations to the generalizability of their results. Of note, all three trials discussed within the “Diagnostic Disparities” and “Undertreatment” sections were conducted within a single health care system. The clinical data found within this health care system may not reflect that of other medical institutions nationwide. It is likely that practice patterns vary geographically as well as across different patient populations within a particular region. Additionally, the reviewed trials lack data regarding patients who may have received AVR at an outside institution after an initial diagnostic echocardiogram within the studied health care system. It is not uncommon for patients to seek care across multiple health care systems (i.e., patients interested in obtaining a second opinion). Furthermore, patients referred to or transferred into a given health care system from outside institutions may wish to undergo definitive treatment closer to home. Data gaps such as these could lead to significant underreporting of AVR utilization.

## Conclusions

**Figure 1:**
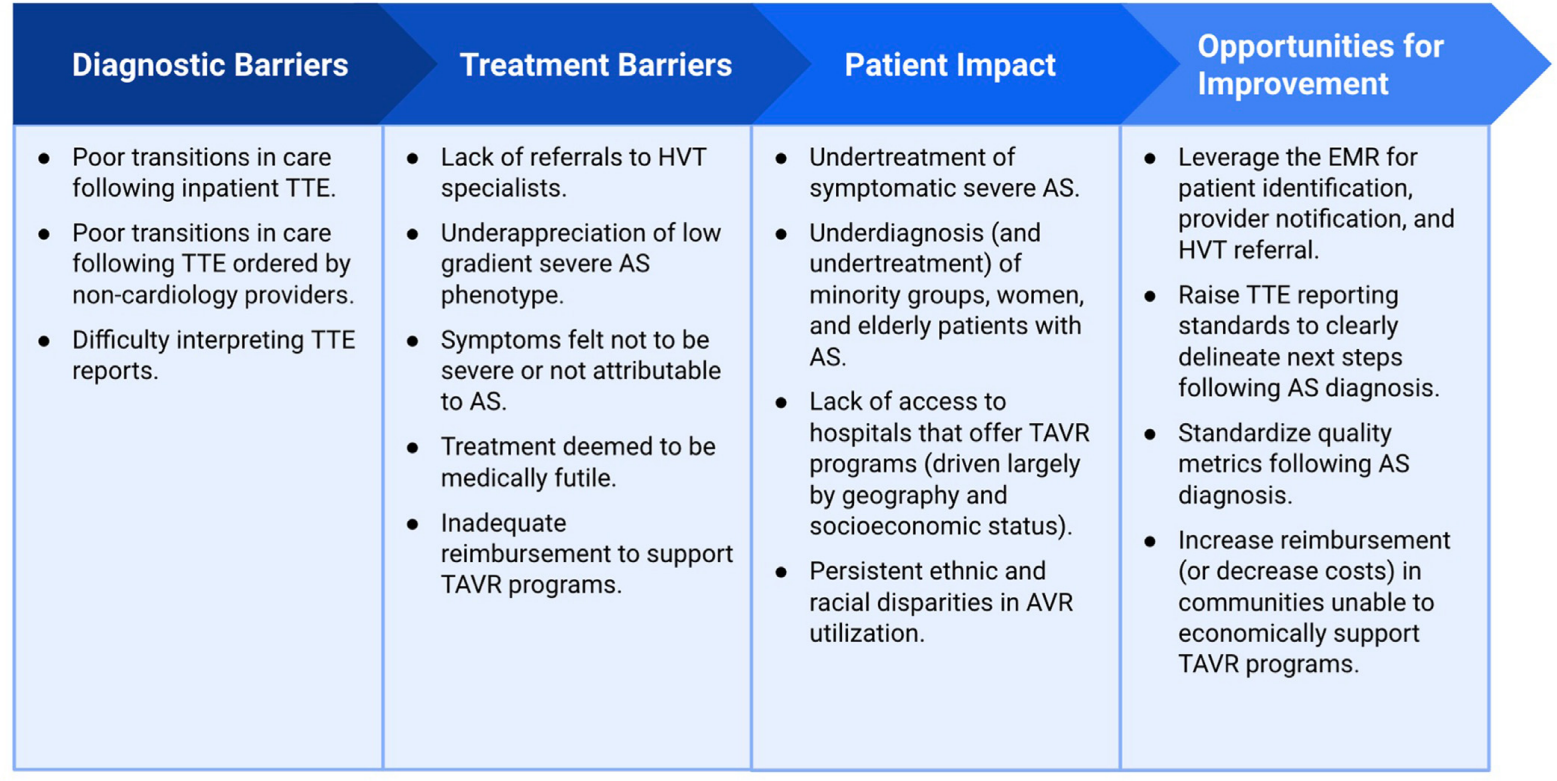
Obstacles and opportunities in the management of AS. Abbreviations: AS, aortic stenosis; AVR, aortic valve replacement; EMR, electronic medical record; HVT, heart valve team; TAVR, transcatheter aortic valve replacement; TTE, transthoracic echocardiogram.

Barriers along the continuum of care for AS can occur at any stage, as summarized in Figure 1. Approximately 1 in 4 patients with either moderate or severe AS is not clinically recognized despite the presence of a diagnostic echocardiogram. The rate of missed diagnosis worsens among underrepresented minority groups and women, and is further exacerbated by reduced access to cardiology specialists and poor transitions in care. Only 50% of patients with symptomatic severe AS undergo AVR within 2 years. One in three patients with symptomatic high-gradient severe AS does not receive AVR. Two in three patients with symptomatic low-gradient severe AS do not receive AVR. While several opportunities exist for the improvement of aortic valve disease management, perhaps most critical is timely referral to a heart valve team specialist whenever AVR is being considered. Initiation of the AS treatment pathway ensures that patients get the opportunity to learn about and potentially receive lifesaving interventions.

## Funding

The authors have no funding to report.

## Disclosure Statement

The authors report no conflict of interest.
